# De-escalating the axilla: oncologic and morbidity outcomes of targeted axillary dissection versus axillary lymph node dissection following neoadjuvant systemic therapy in clinically node-positive breast cancer within a single institution

**DOI:** 10.3389/fonc.2026.1820926

**Published:** 2026-07-30

**Authors:** Skylar Harbour, Michelle Ki, Connie Chen, Victoria Dai, Sheldon Feldman, Ethan Ravetch, Ruth Samson, Maureen McEvoy

**Affiliations:** 1 Albert Einstein College of Medicine, New York, NY, United States; 2 Department of Breast Surgery, Montefiore Medical Center, New York, NY, United States

**Keywords:** axillary de-escalation, breast surgery, lymphedema, neoadjuvant chemotherapy, targeted axillary dissection (TAD)

## Abstract

**Background:**

Axillary lymph node dissection (ALND) has been standard for regional control in clinically node-positive breast cancer, but causes significant morbidity, including lymphedema and arm dysfunction. Targeted axillary dissection (TAD) is a less invasive alternative after neoadjuvant systemic therapy (NST), which can downstage nodal disease. Emerging evidence suggests that TAD offers oncologic outcomes similar to ALND with less morbidity.

**Methods:**

We conducted a retrospective cohort study of patients with clinically node-positive (cN1) breast cancer treated between 2017 and 2025 at the Montefiore Einstein Breast Care Center who received NST followed by surgery. Patients were categorized based on axillary surgical procedure (TAD or ALND) and evaluated for oncologic and morbidity outcomes. Axillary procedure selection was based on both clinical staging and response to neoadjuvant therapy. Morbidity endpoints include lymphedema, upper extremity functional impairment or range of motion limitations, compression sleeve use, rehabilitation referral, and changes in L-Dex scores. Lymphedema was diagnosed by comparing preoperative and postoperative L-Dex scores, with a change of six or more indicating diagnosis. Patient data were collected for a mean follow-up of four years.

**Results:**

Of 256 patients, 136 (53.1%) underwent TAD, 14 SLNB (5.5%), and 106 ALND (41.4%). ALND had higher clinical tumor stages (p = 0.042) and a larger number of nodes removed (4.5 vs 15.3, p < 0.001). Pathologic complete response (pCR) and axillary complete response rates were higher with TAD (pCR:46% vs. 10.4%, p < 0.001; axillary CR: 64% vs. 12.3%). ALND was associated with higher rates of lymphedema (27.4% vs. 12.7%, p = 0.002), upper-extremity impairment (33.9% vs. 18%, p < 0.001), and rehabilitation referral (39.6% vs. 21.3%, p = 0.005). ALND led to a higher mean postoperative L-Dex score change (5.5 vs. 3.2; p = 0.042), reflecting greater lymphatic dysfunction.

**Conclusion:**

In cN1 patients treated with NST, TAD was associated with significantly lower rates of lymphedema and upper extremity morbidity compared to ALND, while also demonstrating higher pathologic and axillary complete response rates. These key findings emphasize the benefit of selective axillary de-escalation with TAD, supporting its role in reducing treatment-related morbidity while maintaining oncologic safety.

## Background

Traditionally, axillary lymph node dissection (ALND) has been regarded as the standard of care for clinically node-positive breast cancer, providing reliable regional control and oncologic safety. However, the morbidity associated with ALND has become increasingly evident, including higher rates of lymphedema, paresthesia, chronic pain, and limited range of motion (1, 2). Breast cancer-related lymphedema (BCRL) is characterized by regional swelling caused by the accumulation of protein-rich lymphatic fluid in the axilla or upper extremity (3). Common symptoms include pain, tightness, reduced upper extremity mobility, and functional limitations that impair activities of daily living (4, 5). Objective assessment of lymphedema is performed using a bioimpedance spectroscopy, which measures the quantity of extracellular fluid in the extremity through low level electrical currents (6). The resulting lymphedema index (L-Dex) compares fluid levels in the at-risk arm with the side that did not receive axillary surgery. The long-term impact of BCRL remains substantial, with reported incidences ranging from 7% to 24.2% following ALND, particularly in patients receiving taxane-based chemotherapy and nodal radiation (7–9). These findings underscore the need for safe axillary de-escalation strategies to minimize treatment-related morbidity without compromising oncologic outcomes (10).

Neoadjuvant systemic therapy (NST) has provided clinically nodal-positive breast cancer patients with the opportunity for nodal downstaging prior to surgery, opening the way for less invasive axillary management. This shift led to the introduction of TAD, developed in response to pivotal trials such as the ACOSOG Z1071 and SENTINA trials (11, 12). Unlike sentinel lymph node biopsy alone, TAD involves the removal of both sentinel lymph nodes and the biopsy-proven positive node that was clipped before NST. This approach improves diagnostic accuracy and reduces false-negative rates compared to SLNB while limiting the overall extent of nodal removal (13). Prior studies have established a direct relationship between the number of nodes excised and the risk of breast cancer-related lymphedema (BCRL), with a prior meta-analysis showing ALND carries a 13% higher risk of lymphedema compared with SLNB (8). Given TAD’s similar amount of nodal removal compared to standard sentinel removal, it presents a promising strategy to preserve oncologic safety while reducing morbidity relative to ALND.

Despite increasing adoption of TAD, comparative data simultaneously addressing both oncologic outcomes and functional morbidity in cN1 patients remains limited. Institutional practice patterns have evolved over time, with changing the adoption of TAD relative to ALND and concurrent changes in systemic and radiation therapy, factors that can confound or modify outcome comparisons. Capturing these temporal dynamics alongside patient, tumor, and treatment-specific covariates is essential to provide contextually valid estimates of comparative effectiveness. Furthermore, ongoing trials such as Alliance A011202 (11) have yet to fully clarify the long-term oncologic safety and quality of life implications in axillary de-escalation. This study aims to assess the oncologic and morbidity outcomes of comparing selective versus complete axillary dissection in cN1 patients treated with NST at a high-volume, single-institution center. We hypothesized that, after adjustment for tumor and treatment factors, TAD provides non-inferior oncologic control with significantly lower morbidity than ALND.

## Methods

This was a retrospective cohort study of all patients treated at the Montefiore Einstein Breast Care Center between 2017 to 2025. Eligible participants included those with biopsy-proven breast cancer staged as clinical node-positive 1 (cN1) by the AJCC 8th edition. cN1 was defined as mobile, ipsilateral level I–II axillary lymph node involvement confirmed by targeted axillary ultrasound and core needle biopsy (14).

Exclusion criteria include a history of prior definitive axillary surgery, cN2–3 disease, or evidence of distant metastasis at diagnosis. All patients received NST, consisting of chemotherapy, endocrine therapy, and/or HER2-directed therapy as indicated by tumor subtype.

A total of 256 patients met inclusion criteria and were categorized by an axillary surgical procedure into two groups: TAD or SLNB, and ALND (levels I-II dissection). All patients undergoing TAD received a radiopaque clip in the biopsy-proven positive node under ultrasound guidance at diagnosis. Nodal metastases were confirmed by core biopsy and targeted axillary ultrasound prior to clip placement. 14 patients initially planned for TAD either lacked clip placement before surgery due to outside or clip loss and thus underwent SNLB instead. Because their clinical and pathologic profiles resembled the TAD cohort, these patients were pooled with the TAD group for subsequent analyses.

Following NST, treatment response was assessed on US, mammography, or MRI and compared with baseline imaging. Response was categorized as complete, partial, no change, or worse. The choice of axillary procedure was determined by both clinical staging and response to neoadjuvant therapy. For patients with clipped nodes, a radioactive iodine-125 seed was placed under ultrasound guidance within 5 days of the surgical procedure. Dual mapping was performed using technetium-99m and/or isosulfan blue, injected into the retroareolar region to identify the sentinel lymph node. Intraoperatively, a gamma probe was used to localize both the I-125–marked target node and technetium-avid sentinel nodes, with radiologic confirmation of the clipped node ex vivo. For TAD, frozen section analysis was performed at the surgeon’s discretion. If the frozen section yielded evidence of isolated tumor cells, micrometastases and/or macrometastases, the result was considered positive, and an ALND was performed. Decisions regarding adjuvant radiation, including regional nodal irradiation, were made by multidisciplinary consensus based primarily on pre-treatment clinical stage and additional clinicopathologic risk features rather than on post-treatment nodal status alone. Consistent with prevailing practice during the study period, many patients who converted to a nodal pCR still received regional nodal irradiation, as response-guided omission of nodal irradiation in this setting was not yet established and remained under prospective investigation (15).

Clinical outcomes assessed included pathological complete response (pCR), residual disease burden, and recurrence, including site and timing. Morbidity outcomes including lymphedema, upper extremity impairment, limited range of motion, and need for rehabilitation therapy were measured. Lymphedema was defined as a change in L-Dex score ≥ 6 from preoperative to postoperative measurements. 7 patients (2.7%) were excluded from lymphedema analysis due to lack of follow-up and L-Dex measurements. Patients were followed for an average of four years, and all relevant clinical data were recorded. All variables were abstracted from the electronic health record (EHR) into a standardized dataset. Data collected included demographic, clinical, radiologic, surgical, pathologic, and treatment data, extracted from operative reports, imaging studies, pathology results, and clinic documentation. Albert Einstein College of Medicine IRB #2023–15017 was approved for this study.

All statistical analyses were conducted using R. Baseline demographics, oncologic and treatment characteristics, and outcomes were summarized descriptively. Continuous variables are reported as means with standard deviations. Normality was assessed using the Shapiro–Wilk test, and group comparisons were performed with two-sample t-tests. Categorical variables are reported as counts (%) and compared with χ² or Fisher’s exact tests, as appropriate. Missing data were handled by complete-case analysis without imputation. Two-sided p<0.05 was considered statistically significant.

## Results

Among the 256 patients evaluated, 211 initially underwent a de-escalated axillary approach (197 TAD, 14 SLNB), while 45 were treated with upfront ALND. Intraoperative frozen section findings or extensive fibrosis led to conversion from TAD to ALND in 61 cases (28.9%); and an additional 8 patients (3.7%) required delayed ALND after final pathology review. This resulted in the final analytic cohort of 150 patients in the TAD group and 106 patients in the ALND group.

Baseline demographics were comparable between cohorts (**Table 1**). The average age was 55.9 ± 13.2 years for TAD and 57.3 ± 13.3 years for ALND. Nearly all patients were female (98%), with no difference in sex distribution. BMI, race, menopausal status, and insurance type also showed no statistical variation.

**Table 1:** Baseline characteristics by surgery type.

	All patients (n= 256)	TAD (n=150)	ALND (n=106)	p-value
Age at Dx (mean ± SD)	56.5 ± 13.2	55.9 ± 13.2	57.3 ± 13.3	0.449
Sex				0.1642†
M	5 (2%)	1 (0.7%)	4 (3.8%)	
F	251 (98%)	149 (99.3%)	102 (96.2%)	
BMI (mean ± SD)	29.5 ± 6.1	29.8 ± 5.9	29 ± 6.3	0.33
Menopause status				0.5854
Pre	98 (38.3%)	56 (37.3%)	42 (39.6%)	
post	150 (58.6%)	91 (60.7%)	59 (55.7%)	
Race (%)				0.9774†
White	98 (38.3%)	58 (38.7%)	40 (37.7%)	
Black	115 (44.9%)	63 (42%)	52 (49.1%)	
Asian	11 (4.3%)	8 (5.3%))	3 (2.8%)	
Other	30 (13.4%)	18 (13.8%)	12 (12.8%)	
Hispanic Ethnicity (%)	101 (39.5%)	64 (42.7%)	37 (34.9%)	0.0989
Interpreter needed (%)	48 (21.4%)	30 (23.1%)	18 (19.1%)	0.5132
Insurance Type (%)				0.5109†
Medicare	94 (36.7%)	54 (36%)	40 (37.7%)	
Medicaid	72 (28.1%)	41 (27.3%)	31 (29.2%)	
Private	89 (34.8%)	54 (36%)	35 (33%)	
Smoking status				0.8103
Never	184 (71.9%)	108 (72%)	76 (71.7%)	
Current	21 (8.2%)	11 (7.3%)	10 (9.4%)	
Former	51 (19.9%)	31 (20.7%)	20 (18.9%)	
HTN	123 (54.9%)	69 (53.1%)	54 (57.4%)	0.1569

† Indicates Fisher Exact Test.

Differences in oncologic features were observed between groups (**Table 2**). Although overall clinical stage distribution was not statistically distinct, ALND patients tended to present higher clinical T stages. Histologic subtypes and tumor locations were comparable between groups; however, hormone receptor status differed significantly, with a greater proportion of HER2-positive and triple-negative breast cancer patients undergoing TAD (p = 0.003).

**Table 2:** Oncology characteristics by surgery type.

	All patients (n=256)	TAD (n=150)	ALND (n=106)	p-value
Previous breast Cancer	11 (4.3%)	7 (4.7%)	4 (3.8%)	1
Clinical stage				0.1293†
I	28 (10.9%)	17 (11.3%)	11 (10.4%)	
II	129 (50.4%)	79 (52.7%)	50 (47.2%)	
III	92 (35.9%)	53 (35.3%)	39 (36.8%)	
IV	7 (2.7%)	1 (0.7%)	6 (5.7%)	
Clin T				*0.0417
I	43 (16.8%)	29 (19.3%)	14 (13.2%)	
II	127 (49.6%)	80 (53.3%)	47 (44.3%)	
III	57 (22.3%)	31 (20.7%)	26 (24.5%)	
IV	29 (11.3%)	10 (6.7%)	19 (17.9%)	
ER/PR/HER2 Status				*0.003
Luminal like (HR+/HER2-)	119 (46.5%)	55 (36.7%)	64 (60.4%)	
HER2+ (HR-)	31 (12.1%)	22 (14.7%)	9 (8.5%)	
Triple Positive (HR+/HER2+)	49 (19.1%)	34 (22.7%)	15 (14.2%)	
TNBC	57 (22.3%)	39 (26%)	18 (17%)	
Histologic subtype				0.09729†
IDC	234 (91.4%)	143 (95.3%)	91 (85.8%)	
ILC	10 (3.9%)	3 (2%)	7 (6.6%)	
Mixed	9 (3.5%)	3 (2%)	6 (5.7%)	
Other	3 (1.2%)	1 (0.7%)	2 (1.9%)	
Clinical Tumor Size (mean)	36.1 + 20.4	34.4 + 19.5	38.5 + 21.3	0.137
Node Size on Imaging (mean)	18.3 + 8.8	19.1 + 9.7	17.2 + 7.2	0.182
Number of abnormal LNs *				0.6958
1	136 (53.1%)	82 (54.7%)	54 (50.9%)	
2	40 (15.6%)	22 (14.7%)	18 (17%)	
3	12 (4.7%)	5 (3.3%)	7 (6.6%)	
4+	68 (26.6%)	41 (27.3%)	27 (25.5%)	
Surgery type				*9.99e-5 †
BCS	128 (50%)	105 (70%)	23 (21.7%)	
Simple/SSM/NSM	100 (39.1%)	45 (30%)	55 (51.9%)	
Modified Radical	28 (10.9%)	0 (0%)	28 (26.4%)	
Reconstruction				*0.0371†
None	179 (69.9%)	106 (70.7%)	73 (68.9%)	
Oncologic reduction	9 (3.5%)	9 (6%)	0 (0%)	
Tissue Expander	11 (4.3%)	6 (4%)	5 (4.7%)	
Direct To Implant	38 (14.8%)	20 (13.3%)	18 (17%)	
Autologous	20 (7.8%)	10 (6.7%)	10 (9.4%)	

† Indicates Fisher Exact Test.

*Abnormal lymph node: a node with morphologically suspicious features (cortical thickening, enlargement, or loss of the normal fatty hilum) on ultrasound, MRI, or mammography at diagnosis.

Surgical approach is also correlated with an axillary approach. All modified radical mastectomy cases underwent ALND, whereas lumpectomy was significantly associated with TAD (p < 0.001). While reconstruction rates were overall similar, only TAD patients underwent oncologic reduction. This was statistically significant (p= 0.037).

Prior to chemotherapy, clip placement in the biopsy-confirmed positive node was performed in 136 (90%) of patients in the SLNB/TAD group and 81 (76.6%) in the ALND group- a difference that was not statistically significant (**Table 3**). Types of systemic therapy (i.e. NAC versus NET), completion rates, dose reductions, and total cycles were similar between groups. However, treatment response favored the TAD group: mean tumor size reduction was significantly greater (66.4%, p < 0.001), and post NST complete response rates were higher (p < 0.001). Postoperatively, endocrine therapy was more common among ALND patients (p = 0.00139), while maintenance HER2-targeted therapy was more common following TAD (p = 0.0247). Overall, 81.6% of patients received adjuvant radiation therapy, with comparable proportions of total radiation and nodal radiation between groups (67.3% vs. 72.6%). ALND patients underwent a greater mean number of fractions per treatment session (21 ± 6.1, p = 0.0113) and were significantly more likely to receive chest wall radiation (57.5%, p <0.001).

**Table 3:** Treatment characteristics by surgery type.

	All patients (n= 256)	TAD (n= 150)	ALND (n= 106)	p-value
Clip retrieved(%)	176 (68.8%)	136 (90%)	81 (76.6%)	.1566
NST Type				0.283
NAC	203 (79.3%)	124 (82.7%)	79 (74.5%)	
NET	35 (13.7%)	17 (11.3%)	18 (17%)	
Both	18 (7%)	9 (6%)	9 (8.5%))	
NAC regimen				0.2208†
T/AC, TC, AC	116 (45.3%)	60 (40%)	56 (52.8%)	
TCHP	74 (28.9%)	51 (34%)	23 (21.6%)	
CMF	6 (2.3%)	3 (2%)	3 (2.8%)	
Keynote	11 (4.3%)	9 (6%)	2 (1.9%)	
Other	14 (5.4%)	10 (6.6%)	4 (3.7%)	
NET regimen				0.1994†
Aromatase Inhibitor	42 (16.4%)	21 (14%)	21 (19.8%)	
Tamoxifen	6 (2.3%)	1 (0.7%)	5 (4.7%)	
Other	7 (2.7%)	5 (3.3%)	2 (1.8%)	
NST completed (%)	219 (85.5%)	133 (88.7%)	86 (81.1%)	0.2651
Dose reduction (%)	46 (18%)	30 (20%)	16 (15.1%)	0.1478
Mean % tumor reduction post NST	57.6 ± 40.3	66.4 ± 36.8	42.4 ± 41.2	*9.64e-5
Clinical response post NST				*9.99e-05†
complete	90 (35.2%)	76 (50.7%)	14 (13.2%)	
partial	123 (48%)	68 (45.3%)	55 (51.9%)	
no change	35 (13.7%)	6 (4%)	29 (27.4%)	
worse	8 (3.1%)	0 (0%)	8 (7.5%)	
Adjuvant RT (%)	209 (81.6%)	123 (82%)	86 (81.1%)	0.8546
RT regimen				*0.001
Chest Wall	85 (33.2%)	24 (16%)	61 (57.5%)	
Nodes	178 (69.5%)	101 (67.3%)	77 (72.6%)	
IMN	86 (33.6%)	37 (24.7%)	49 (46.2%)	
SCV	148 (57.8%)	78 (52%)	70 (66%)	
Axilla	140 (54.7%)	80 (53.3%)	60 (56.6%)	
Mean Fx	19.6 ± 6.3	18.7 ± 6.4	21 ± 6.1	*.0113
Mean Gy	50.1 ± 8	49.6 ± 9	50.7 ± 6.3	0.366
Adjuvant ET (%)	139 (62.1%)	69 (53.1%)	70 (74.5%)	*0.00139
ET regimen				1†
Aromatase Inhibitor	107 (47.8%)	53 (40.8%)	54 (57.4%)	
Tamoxifen	22 (9.8%)	11 (8.5%)	11 (11.7%)	
Other	9 (4%)	5 (3.8%)	4 (4.3%)	
Adjuvant CT (%)	37 (16.5%)	18 (13.8%)	19 (20.2%)	0.7832
CT regimen				0.3986†
T/AC	15 (6.7%)	5 (3.8%)	10 (10.6%)	
THP	6 (2.7%)	4 (3.1%)	3 (3.2%)	
TDM1	5 (2.2%)	4 (3.1%)	1 (1.1%)	
Capecitabine	10 (4.5%)	5 (3.8%)	5 (5.3%)	
Maintenance				*0.0247†
HER2	73 (32.6%)	48 (36.9%)	25 (26.6%)	
Pembrolizumab	13 (5.8%)	11 (8.5%)	2 (2.1%)	
CDK4/6 Inhibitor	9 (4.0%)	4 (3.1%)	5 (5.3%)	
PARP Inhibitor	1 (0.4%)	0 (0%)	1 (1.1%)	

† Indicates Fisher Exact Test.

Significant differences were observed in pathologic outcomes between the TAD and ALND groups (**Table 4**). Pathologic complete response (pCR) was significantly higher in TAD patients compared to ALND patients (46% vs. 10.4%, p<0.001), and axillary complete response was significantly greater in TAD patients (64% vs. 12.3%, p<0.001). Patients undergoing ALND had significantly more nodes removed (mean, 15.3 vs. 4.2; p < 0.001) and a higher mean number of positive nodes (5.7 vs. 0.6; p < 0.001). Overall recurrence and average follow-up times were statistically insignificant.

**Table 4:** Oncologic outcomes by surgery type.

	All patients (n=256)	TAD (n= 150)	ALND (n= 106)	p-value
Progression to ALND				
Intraoperatively delayed			61 (57.5%) 8 (8.5%)	
Pathologic T				*9.999e-5 †
0/is	97 (37.9%)	78 (52%)	19 (17.9%)	
I II	88 (34.4%) 50 (19.5%)	55 (36.6%) 15 (10%)	33 (31.1%) 35 (33%)	
III	16 (6.2%)	2 (1.3%)	14 (13.2%)	
IV	5 (2%)	0 (0%)	5 (4.7%)	
Pathologic N				*9.99e-5 †
0	106 (41.4%)	91 (60.7%)	15 (14.2%)	
0 (i+)/mi	25 (9.8%)	21 (14%)	4 (3.8%)	
I	68 (26.6%)	34 (22.7%)	34 (32.1%)	
II	40 (15.6%)	3 (2%)	37 (34.9%)	
III	17 (6.6%)	1 (0.7%)	16 (15.1%)	
Nodes Removed (mean ± SD)	8.6 ± 8.2	4.2 ± 2.7	15.3 ± 8.8	*2e-16
Positive Nodes (mean ± SD)	2.8 ± 5.2	0.6 ± 1.4	5.7 ± 6.8	*9e-15
Sentinel Nodes Removed (mean ± SD)	3 ± 2.4	3.3 ± 2.2	2.6 ± 2.7	*0.0299
Positive Nodes (mean ± SD)	1.03 ± 1.63	0.6 ± 1.4	1.9 ± 1.7	*1.07e-07
Axillary Reverse Mapping (%) Blue nodes removed (mean ± SD)	76 (33.9%) 0.8 ± 0.9	35 (26.9%) 1 ± 1	41 (43.6%) 0.5 ± 0.6	*0.01384 *0.0471
Axillary complete response (%)	109 (42.5%)	96 (64%)	13 (12.3%)	*2.079e-14
Total pathologic CR (%)	80 (31.2%)	69 (46%)	11 (10.4%)	*0.001
Recurrence (%)	23 (8.9%)	6 (4%)	12 (11.3%)	0.1007
Local (%)	2 (0.7%)	1 (0.7%)	1 (0.9%)	
Nodal (%)	7 (2.7%)	2 (1.4%)	3 (1.8%)	
Distant (%)	14 (5.5%)	3 (2.1%)	8 (7.5%)	
Mean Time to Recurrence (months)	28.6 ± 19.9	29.7 ± 19.3	28.1 ± 20.5	0.825
Mean follow up time	42.7 ± 27.3	44.4 ± 26.9	40.3 ± 27.7	0.267

† Indicates Fisher Exact Test.

Postoperative morbidity outcomes favored TAD (**Table 5**). Patients undergoing ALND experienced higher lymphedema rates (27.4% vs. 12.7%, p=0.002), upper extremity impairment or range of motion limitation (33.9% vs. 18%, p<0.001), use of compression sleeves (34.9% vs. 16.9%, p=0.004), and rehabilitation referrals (39.6% vs. 21.3%, p=0.005). Moreover, the mean postoperative change in L-Dex score was significantly greater among ALND patients (5.5 ± 4.3 vs. 3.2 ± 4.6, p = 0.042), reflecting more pronounced lymphatic dysfunction. These differences persisted on multivariable analysis. In logistic models adjusting for tumor subtype, clinical stage, and response to NST, surgical approach remained an independent predictor: ALND was associated with approximately 2.7 times higher odds of lymphedema compared with TAD (OR 2.70, 95% CI 1.35–5.58, p = 0.006). This association remained significant in models additionally accounting for nodal burden and reconstruction, and none of the examined covariates- including age, BMI, axillary reverse mapping (ARM), nodal radiation, and reconstruction- demonstrated an independent association with lymphedema.

**Table 5:** Postoperative morbidity outcomes.

	All patients (n=256)	TAD (n= 150)	ALND (n= 106)	p-value
Lymphedema (%)	48 (18.8%)	19 (12.7%)	29 (27.4%)	*0.002
Upper Extremity Impairment or ROM Limitation (%)	63 (24.6%)	27 (18%)	36 (33.9%)	*0.0007
Use of Compression Sleeve (%)	62 (24.1%)	25 (16.9%)	37 (34.9%)	*0.004
Referral to Rehab/PT (%)	74 (29%)	32 (21.3%)	42 (39.6%)	*0.005
L-Dex Score Post-op (mean)	-0.2 ± 8.4	-0.5 ± 6.8	0.3 ± 10.5	0.716
Change in L-Dex Score (mean)	4.1 ± 4.6	3.2 ± 4.6	5.5 ± 4.3	*0.042

The extent of axillary surgery showed a comparable relationship. When the number of nodes removed was modeled in place of surgical group, each additional node removed was associated with 7% higher odds of lymphedema (p = 0.002 corresponding to approximately 38% increase per 5 nodes), and node count added significant predictive value beyond the adjusted covariates (p = 0.001). In contrast, no independent association with surgical approach or extent of nodal surgery was identified for upper-extremity pain or decreased range of motion after adjustment. In models examining nodal radiation and its interaction with surgical approach, nodal radiation was not independently associated with lymphedema (p = 0.97) or upper-extremity pain (p = 0.69), and the nodal radiation by procedure interaction was non-significant (p = 0.89 for lymphedema).

## Discussion

Targeted axillary dissection (TAD) has become increasingly utilized in the management of clinically node-positive (cN1) breast cancer (16). This less invasive approach offers a meaningful reduction in postoperative morbidity by limiting the extent of nodal excision compared to ALND, thereby improving patients’ long-term quality of life. Furthermore, by utilizing node clipping prior to NST, TAD enhances staging precision and reduces false negative rates relative to sentinel lymph node biopsy alone (13). Our study adds to the growing body of evidence by simultaneously evaluating oncologic control and functional morbidity among cN1 patients undergoing TAD vs ALND. Our findings provide a comprehensive assessment of how selective axillary de-escalation can preserve oncologic safety while minimizing treatment-related morbidity in chemotherapy-responsive disease.

### Oncologic outcomes

Our findings indicate that TAD selection largely depends on response to NST. Patients in the TAD group demonstrated higher rates of clinical response on preoperative imaging compared to those undergoing ALND (50.7% vs 13.2%), with intraoperative pCR rates of 46% vs. 10.4% (**Table 4**). Notably, HER2+ and TNBC patients were more frequently represented in the TAD cohort, consistent with the known higher responsiveness of these subtypes to NST relative to ER/PR+ tumors (17).

Despite differences in response rates, the oncologic outcomes were comparable between groups, with no significant difference in recurrence observed. These results underscore the oncologic safety of TAD when applied based on tumor biology and treatment response. Importantly, although ALND yielded a greater number of nodes removed and higher nodal positivity, this did not confer additional benefit in terms of recurrence, suggesting that removal of additional nodes may not improve oncologic outcomes in the context of effective systemic therapy. Our findings align with prior studies, including the SenTa and I-SPY2 trials, which similarly demonstrate low recurrence rates with TAD comparable ALND, even among initially node-positive patients (18, 19). In the prospective multicenter SenTa study, TAD alone after NST achieved a high clipped node detection rate and a low FNR, with axillary recurrence and disease-free survival comparable to TAD combined with ALND, supporting the oncologic feasibility of omitting ALND in selected responders (16). The reproducibility of this approach across localization platforms is further supported by the Massachusetts General Hospital experience reported by Laws et al., in which TAD using non-radioactive wireless localizers achieved a 91% clipped-node retrieval rate and spared completion ALND in 85% of patients who converted to negative nodal status (20). Together with our radioactive seed technique, these data reinforce the generalizability of response-guided axillary de-escalation.

### Morbidity

ALND was associated with significantly higher morbidity compared to TAD, including a 14.7% higher rate of lymphedema (27.4% vs. 12.7%, **Table 5**). Objective measurements supported this disparity, with ALND patients demonstrating greater postoperative changes in L-Dex scores. These findings are consistent with prior studies, including the Neosenti-Turk/MF-18–03 and AMAROS trials, which reported differences in lymphedema rates between the groups of 15% and 12.6%, respectively (21, 22). While previous studies have reported higher rates of lymphedema in ALND patients who also received nodal radiation (23), ALND remained independently associated with lymphedema on multivariate analyses adjusting for nodal radiation, which was not itself a significant predictor. Moreover, the proportion of patients receiving nodal radiation was similar across ALND and TAD cohorts. Overall, these results suggest that the higher lymphedema rates are primarily attributable to the ALND procedure rather than differences in adjuvant treatments. Although regional node irradiation is a recognized contributor to lymphedema risk, in our cohort it was balanced between surgical groups and was not identified as an independent predictor in multivariable analysis, nor did its interaction with surgical approach reach significance. The addition of nodal radiation was not associated with a meaningful change in lymphedema rates, consistent with the claim that axillary dissection rather than radiation is the predominant driver of morbidity outcomes. This is supported by the NSABP B-51/RTOG 134 trial, which found no oncologic benefit of regional nodal irradiation in patients converting to ypN0 after NST, suggesting that radiation de-escalation in responders may further reduce treatment-related morbidity without compromising oncologic outcomes (15).

ALND was also associated with higher incidences of upper extremity pain and limited range of motion, consistent with existing literature demonstrating increased swelling, paresthesia, and decreased shoulder mobility (24–28). Reflecting higher BCRL rates, ALND had greater utilization of compression sleeves (34.9%) and referrals for physical therapy (39.6%), suggesting a greater need to manage postoperative morbidities. These differences parallel the broader literature on axillary morbidity. In a systematic review and meta-analysis of 67 studies, Che Bakri et al. reported that ALND is associated with approximately a 13.7% higher rate of lymphedema and a 24.2% higher prevalence of pain compared with sentinel lymph node biopsy, closely mirroring the differences observed between our ALND and TAD cohorts (8). The higher rates of rehabilitation referral and compression sleeve use in our ALND group reflect this greater functional burden. In a systematic review of postoperative physical therapy after breast cancer treatment, De Groef et al. found that physical therapy interventions can effectively reduce pain and improve shoulder range of motion, underscoring the value of early identification and referral of higher risk patients such as those undergoing ALND (29). Notably, some patients reported discomfort and swelling despite no formal lymphedema diagnosis via L-Dex calculations, indicating subclinical lymphatic disruption. These high rates of functional impairment highlight the impact on quality of life, as chronic BCRL can limit daily activities, increase healthcare costs, and negatively affect mental health (30, 31). In contrast, patients undergoing TAD experienced markedly fewer complications, reinforcing the benefit of targeted, less invasive axillary approaches in modern breast cancer care.

### Limitations

Our limitations include the study’s retrospective design and single-institution selection for participants our analysis is subject to selection bias, and although we adjusted for tumor subtype, clinical stage, treatment response, and additional clinical variables in multivariable analysis, residual confounding from unmeasured factors cannot be excluded. The mean follow-up of approximately four years is relatively short for node-positive disease, in which recurrences continue to accrue over a longer period, and the study was not powered to formally establish oncologic equivalence or non-inferiority. The non-significant difference in recurrence should therefore be regarded as absence of a detected difference rather than proof of equivalent oncologic outcomes, particularly given the baseline differences in tumor biology, clinical stage, and treatment response between the TAD and ALND cohorts. These imbalances reflect the response-guided application of TAD and may independently influence recurrence risk despite multivariable adjustment. Additionally, L-Dex scores recorded postoperatively were missing in a few patient reports, which could have potentially skewed results, despite having a clear number of lymphedema diagnoses as determined by ICD codes. The prevalence of lymphedema reduced ROM, and other functional impairments may be under-reported as the outcomes measured were by diagnoses and not subjective tools such as questionnaires. Prospective incorporation of validated instruments such as the QuickDASH for upper extremity function would strengthen morbidity ascertainment in future studies. Despite these limitations, our study demonstrated a significant difference in functional impairments following ALND and TAD.

## Conclusion

Our findings add to the growing evidence that axillary de-escalation can spare patients from long-term morbidities associated with breast cancer treatment, particularly lymphedema. In appropriately selected, chemotherapy-responsive cN1 patients, TAD was associated with significantly lower morbidity than ALND, with no significant difference in recurrence over the available follow-up. The adoption of TAD has the potential to improve survivorship quality while appearing to preserve short-term oncologic control. These results have important clinical implications, supporting a wider adoption of TAD in clinical practice and reinforcing a shift in clinical guidelines towards de-escalated axillary surgery. Ultimately, this approach may lead to improved patient outcomes by minimizing treatment-related complications.

## Data Availability

The raw data supporting the conclusions of this article will be made available by the authors, without undue reservation.

## Supplementary material

Supplementary Figure 1 Flowchart of group selection.
